# Trichostatin A Inhibits Cytokines Released in LPS-Induced THP-1 Cells *via* Downregulating Deacetylated-Syntaxin 17 and Promoting Autophagosome-Lysosome Fusion

**DOI:** 10.33549/physiolres.935799

**Published:** 2026-08-01

**Authors:** Le SANG, Ran ZANG, Zewei YU, Xia GONG, Yunlei HUANG, Xingyu JIN, Jian SUN

**Affiliations:** 1 Department of Pulmonary and Critical Care Medicine, Shaoxing People’s Hospital, Shaoxing, China; 2 School of Medicine, Shaoxing University, Shaoxing, China; 3 Faculty of Information Science and Technology, Universiti Kebangsaan Malaysia, Bangi, Selangor, Malaysia

**Keywords:** Sepsis, Histone deacetylase 2, Deacetylated STX17, Cellular autophagy, Autophagosome-lysosome pathway, Cytokines

## Abstract

Sepsis is a complicated disorder caused by infection, which may trigger various symptoms. Lipopolysaccharides (LPS) are among the primary pathogens which can stimulate host’s immunoreaction. In the late stage of sepsis, the patients’ immune system is significantly suppressed, thus the mortality increases. Notably, autophagy is pivotal during sepsis, in which autophagosome-lysosome fusion mediated by syntaxin 17 (STX17) is a key step. Previous studies have shown that histone deacetylase 2 (HDAC2) deacetylated STX17 to promote autophagosome-lysosome fusion. Trichostatin A (TSA) as a HDAC inhibitor (HDACi) has the capability of inhibiting histone deacetylase activity, while such an effect can be reversed by ITSA-1. This time LPS was used to stimulate human monocyte-macrophages (THP-1 cells) to establish the inflammatory cell model. TSA and ITSA-1 were administrated. The cell proliferation was examined by WST-1. Cytokines such as TNF-α and IL-6, as well as HDAC2 activity and deacetylated STX17 (DA-STX17) expression were detected by ELISA. Also, autophagy-related proteins such as P62 and microtubule-associated protein light chain 3 (LC3) were detected by Western Blotting (WB), and autophagosome-lysosome fusion was observed by fluorescence assay. Cell apoptosis was measured by flow cytometry. Besides, network pharmacology analysis was conducted. The data showed that TSA inhibited HDAC2 activity, and remarkably downregulated DA-STX17 expression, accompanied by the lower expression of P62 and LC3 II/I. Decreased DA-STX17 promoted autophagosome-lysosome fusion, and inhibited TNF-α secretion. Furthermore, network pharmacology analysis revealed an inner relationship concerning HDAC2 between TSA and sepsis. Therefore, TSA can be considered a potential drug to cure sepsis-related diseases.

## Introduction

Sepsis caused by the infection of Gram-negative bacteria is a systemic pathophysiological disorder, which is manifested as various physiological and pathological abnormalities. Sepsis is characterized by a series of inflammatory reactions, including the activation of immunocytes and release of pro-inflammatory cytokines, which may lead to multiple complications such as dysglycemia, hypotension, and septic shock. Therefore, sepsis poses significant challenges to social healthcare systems and seriously affects the quality of patients’ lives [1–4].

LPS, an active component in the cellwall of Gram-negative bacteria, plays a pivotal role in sepsis. LPS binds to specific receptors such as toll-like receptors (TLRs) to initiate a range of signal transduction processes and release cytokines [5, 6]. Properly secreted cytokines have functions such as chemotaxis for phagocytic cells to clear pathogens. However, excessive immunoreactions may trigger systemic inflammatory response syndrome (SIRS), leading to severe damage to the host.

It was believed that in addition to widespread inflammatory response, hyperimmunity was the fundamental cause of death in sepsis patients. However, it was found that partial patients who survived in the initial phase of excessive inflammatory response may subsequently experience prolonged illness. These patients have an increased risk of opportunistic infection due to the exhaustion of immunocytes, characterized by suffering from repetitive nosocomial infection and high protein catabolism. The syndrome is known as “Persistent Inflammation Immunosuppression, and Catabolic Syndrome (PICS)”. Patients with PICS have a mortality rate as high as 45 % to 71 % [7]. Notably, sepsis patients show a remarkable decrease in lymphocyte and cytokine levels at the late stage, suggesting that immunosuppression might be the fundamental factor of death.

Autophagy is a conserved metabolic mechanism ubiquitous in eukaryotes, functioning to precisely degrade damaged organelles via lysosomal activation. Normally, autophagy clears accumulated cargo to ensure cellular function [8].

However, in diseases such as sepsis, the role of autophagy becomes multifaceted. Studies have revealed that the protective effect of autophagy [9], for example, autophagy reduces intracellular reactive oxygen species (ROS) and mitigates oxidative stress damage [10]. Yet, some studies have found that inhibiting autophagy reduces mortality in septic rats [11]. Thus, the role of autophagy in sepsis requires further elucidation.

LC3 and P62 are representative proteins involved in autophagosome formation. LC3 is cleaved at the carboxyl terminus by autophagy-related gene (Atg) 4 to form cytoplasmic LC3 (LC3-I). LC3-I subsequently undergoes ubiquitination involving Atg7 and Atg3 to couple with phosphatidyl-ethanolamine (PE) and transforms into membrane LC3 (LC3-II). Thus, the LC3 II/I ratio reflects the degree of autophagic flux. Additionally, P62 is also a widely studied autophagy substrate that can bind ubiquitinated proteins, and then be degraded via the autophagosome-lysosome pathway [12]. It is known that when autophagic flux is inhibited, P62 expression increases [13].

STX17 is a key factor in autophagosome-lysosome fusion. STX17 binds to SNAP29 protein to form a t-SNARE complex. The complex then interacts with the VAMP8 protein on the lysosomal membrane to form a SNARE complex. Mediated by the SNARE complex, autophagosomes fuse with lysosomes to release hydrolytic enzymes to degrade the cargo within autophagosomes [14, 15]. Studies have also shown that autophagosome-lysosome fusion is significantly reduced after STX17 knockdown [16]. Additionally, studies have shown that high expression of HDAC2 increased the deacetylation of STX17, promoting autophagosome-lysosome fusion. Conversely, downregulating HDAC2 promotes STX17 acetylation, reducing the affinity between STX17 and SNAP29, and thus impairing the autophagosome-lysosome pathway [15].

TSA, as a classic HDACi, is widely used in scientific research. It exhibits diverse biological activities due to its ability to regulate gene expression and influence cell cycle progression. Generally, HDACi inhibits histone deacetylase activity in a dose-dependent manner [17], and regulates cellular biofunctions by modulating downstream proteins such as STX17.

ITSA-1, a common TSA inhibitor, is known to reverse the biological effects of TSA. For instance, ITSA-1 can rescue TSA-impaired cell proliferation and migration [18]. Furthermore, ITSA-1 can re-upregulate the HDAC expression, which was reduced by TSA. However, the precise function and mechanism of TSA in the LPS-induced septic model remain unknown. In the present study, we used TSA and ITSA-1 to therapeutically intervene in the inflammatory cell model, to search for novel methods of treating sepsis clinically.

## Materials and Methods

### Drugs and chemicals

LPS was obtained from Sigma Chemical Company (St. Louis, MO, USA). TSA was purchased from MCE (Shanghai, China), and ITSA-1 was obtained from Topscience (Shandong, China). LPS was dissolved in double-distilled water, while TSA and ITSA-1 were dissolved in dimethyl sulfoxide (DMSO) according to instructions. Primary antibodies used in the research include rabbit monoclonal/polyclonal anti-HDAC2 (MCE, Cat. No. HY-P80151), anti-P62 (MCE, Cat. No. HY-P80899), anti-LAMP1 (MCE, Cat. No. HY-P80206A), anti-LC3 (ORIGENE, Jiangsu, China, Cat. No. RC220602), and anti-STX17 (ORIGENE, Cat. No. TA382164). Anti-β-actin (Cat. No. 4967) and the secondary antibody (Cat. No. 76126) were purchased from Cell Signaling Technology (Danvers, MA, USA). All drugs and chemicals were stored at −20 °C.

### Cell culture and drug intervention

THP-1 cells were purchased from Haoxin Biotech Company (Zhejiang, China). The cells were cultivated in RPMI-1640 medium added with penicillin and streptomycin (BioChannel Biotechnology, Nanjing, China), and 15 % FBS at 37 °C in a humidified atmosphere with 5 % CO_2._ THP-1 cells at a density of 5×10^5^/ml were planted in well plates, and administrated with drugs. After simultaneously treating with LPS (10 μg/ml), TSA (400 nm), and ITSA-1 (50 μm) for 24 h, supernatants were harvested to measure cytokines, and cell precipitates were used for further experiments.

### WST-1 assay

THP-1 cells were planted into the 6-well plate and administrated with LPS, TSA, and ITSA-1 at the aforementioned concentrations. The cell suspension was transferred into the 96-well plates as 100 μl per well, and 10 μl of WST-1 reagent (Sigma Chemical Company) was added into each well. After incubation at 37 °C for 30 min and 24 h, the optical density was measured at a wavelength of 450 nm. Each experimental administration was repeated in at least 3 wells. Cell proliferation levels were calculated as the optical density at 24 h divided by the mean optical density at 30 min of the control group.

### Enzyme-linked immunosorbent assay (ELISA)

In the present study, TNF-α and IL-6 ELISA kits were purchased from NeoBioscience Technology Company (Shenzhen, China). HDAC2 Activity kits were obtained from Biolite (New York, USA), and DA-STX17 kits were purchased from Shanghai Ding Biological Technology (Shanghai, China). After 24 h of administration, the collected supernatants were placed on ice, and ELISA was performed immediately. A minimum of triple duplicate wells were set up, and the optical density of final liquid was measured at 450 nm using the microplate reader (Thermo Scientific, Shanghai, China).

### Real-time quantitative polymerase chain reaction (RT-qPCR)

The THP-1 cells in each group were treated with the aforementioned drugs and harvested by centrifugation. Then the RNA of cells was extracted using universal RNA Purification Kit (EZBioscience, Jiangsu, China) with additional anhydrous ethanol. Reverse transcription was conducted using PrimeScript™ RT Master Mix Kit (Takara, Kyoto, Japan), and RT-qPCR amplification was performed using the TB Green® Premix Ex Taq™ Kit (Takara) and a Light Cycler PCR system (Cobas Z 480, Beijing, China). The relative RNA expression was calculated as 2^(-ΔΔCT). Primer sequences were designed on the NCBI website and approved in PrimerBank. The primer of STX17 was purchased from Tsingke Biology (Beijing, China), and the housekeeping gene was chosen as ACTB gene (Beyotime, Shanghai, China, Accession No. NM_001101). The primer sequences of STX17 gene used in the study were as 5′-3′: CATGACTGTTGGTGGAGCATTTC, 3′-5′: GCTTCT AAGGTTTCCCACGATTC.

### Western blotting analysis

LPS-intervened cells were cleaved by RIPA buffer to collect proteins, and the protein concentration was quantified by BCA Kit (Beyotime). Protein samples were electrophoretically separated on SDS-PAGE gels, and transferred to PVDF membrane (Millipore, USA). Then PVDF membrane was blocked with QuickBlock (Beyotime) for 15 min and incubated with primary antibodies at 4 °C overnight. Then the PVDF membrane was incubated with the secondary antibody for 30 min at room temperature. After triple washing with TBST, protein bands were exposed to the ECL detection machine (Tanon, Shanghai, China), and protein density was analyzed by ImageJ-win64 (NIH, Germany). Primary antibodies were diluted 1:1000~5000, and the secondary antibody was diluted 1:5000~10000.

### Ad-mCherry-GFP-LC3B transfection

THP-1 cells were collected by centrifugation and incubated with increasing volumes of Ad-mCherry GFP-LC3B adenovirus (Beyotime) for 15 min to enhance cell-virus contact. Afterwards, the cells were placed into the 6-well plate. To determine the optimal Multiplicity of Infection (MOI), the transfected cells were observed using the fluorescent inverted microscope (OPLENIC Pro Ver.1.92, Hangzhou, China) after 24 h. The MOI used in the study was ultimately 500. The above transfection protocol was repeated with new THP-1 cells, followed by administration of the aforementioned Drugs. Transfected THP-1 cells were observed under a fluorescence inverted microscope, and images were captured by OPLENIC Pro Ver.1.92.

### Annexin V-7AAD apoptosis kit

THP-1 cell apoptosis was detected using the Annexin V-APC/7-AAD Apoptosis Kit (Elabscience, Hubei, China). After 24 h of treatment, cells were washed twice with PBS and then resuspended in Binding Buffer. Then apoptotic dyes, Annexin V and 5 μl of 7-AAD, were added. After gently vortexing cells were incubated for 15 min in the dark, and then were analyzed immediately using Beckman CytoFLEX (Shanghai, China). Apoptosis levels were quantified as the percentage of Annexin V-positive or Annexin V&7-AAD-positive cells.

### Statistical analysis

The data from a clinical trial (ID: 11480738) were collected from the Gene Expression Omnibus (GEO) public database. Each experiment was repeated for a minimum of triple times (n≥3). Original data were input into GraphPad Prism 10.1.2 (San Diego, CA, USA), and the results were presented as the means±standard deviations (S.D.). Data with normal distribution and homogeneity of variance were analyzed by *t*-test or ANOVA. A value of P < 0.05 was considered statistically significant.

### Network pharmacology analysis of TSA

#### Acquisition of TSA structure and action targets

The 2D and 3D structures and possible therapeutic targets of TSA were acquired from public databases, such as PubChem, Protein Bank Database (PBD), SwissTargetPrediction, and DrugBank.

### Searching for therapeutic targets for sepsis

Therapeutic targets related to sepsis were obtained from GeneCards, DISGENET, and Online Mendelian Inheritance in Man (OMIM) by searching terms “sepsis”. All duplicate targets were automatically removed by the software during subsequent processes.

### Acquisition of intersection genes

The action targets of TSA and therapeutic sites of sepsis were imported into the Venn online website to identify overlapping targets, which were seem as feasible therapeutic targets of TSA in the cure of sepsis.

### Protein-protein interaction (PPI) network analysis

The overlapping protein targets were input into GeneMANIA online tool to establish the PPI network. The key proteins were marked red.

### Functional enrichment analysis

The Gene Ontology (GO) enrichment analysis was to elucidate the underlying mechanisms of TSA in sepsis treatment using the DAVID database. The significance threshold was manually set at P < 0.05. The top 10 enriched GO terms were visualized using RStudio 4.5.2.

### Molecular docking

Potential interactions among autophagy-related proteins as the HDAC2-STX17 axis is the key were checked using molecular docking. Receptor and ligand structures were downloaded from the PubChem and PDB databases. Protein structures were optimized through minimizing steric energy (Chem3D), as well as removing water molecules and organics (PyMOL). Semi-flexible docking was performed by Autodock_Vina 4.2.6, and the positive binding conformations were presented using PyMOL.

## Results

### ITSA-1 remarkably promoted the THP-1 cell proliferation inhibited by LPS

**Fig. 1:**
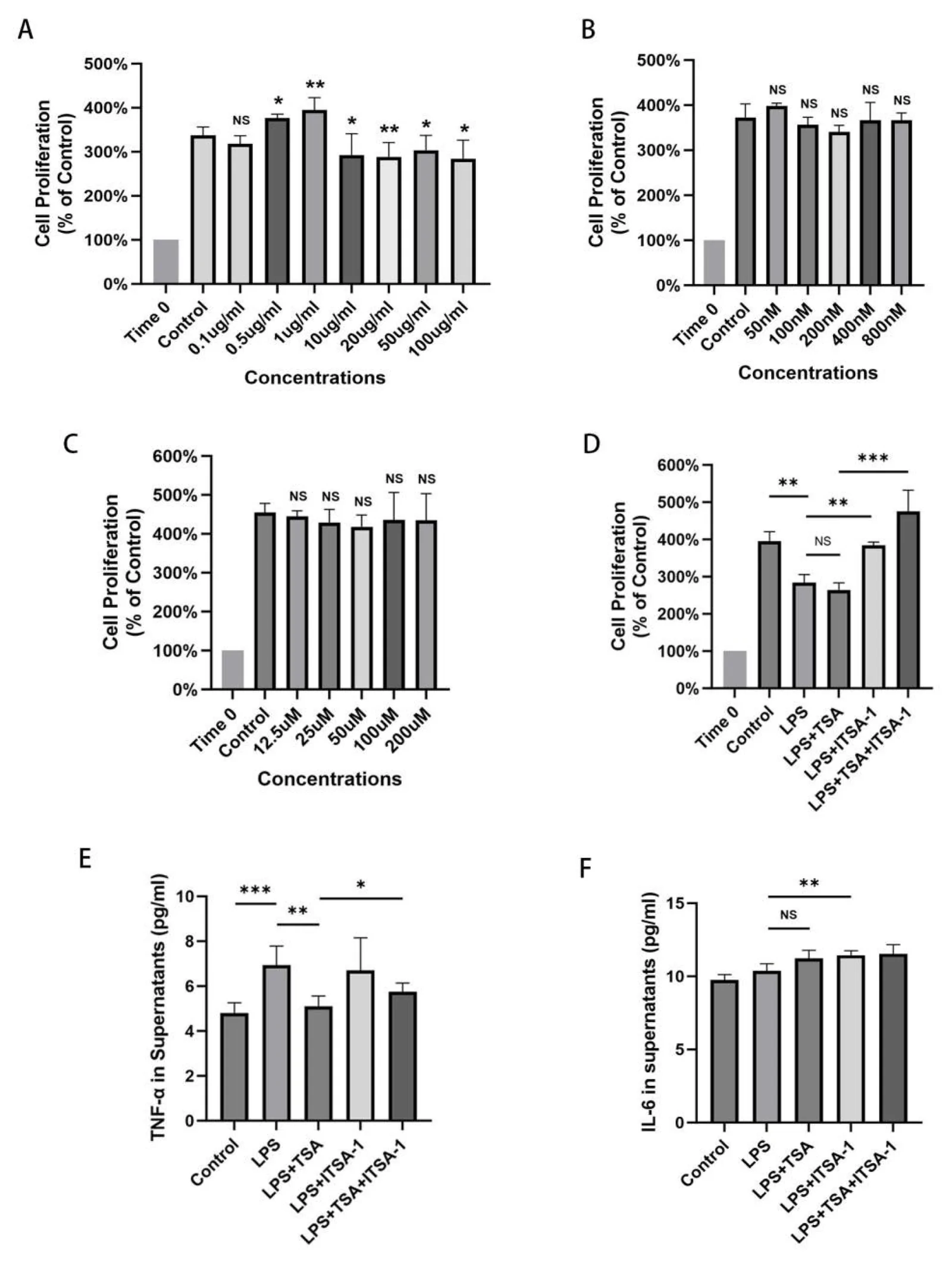
The levels of proliferation and pro-inflammatory cytokines in THP-1 cells intervened with LPS, TSA, and ITSA-1 for 24 h (**A**) Concentration gradient test of LPS (**B**) Concentration gradient test of TSA. (**C**) Concentration gradient test of ITSA-1 (**D**) Cell proliferation levels under different drug interventions (**E**) TNF-α concentration in cell supernatants (**F**) IL-6 concentration in cell supernatants. *P < 0.05, **P < 0.01, ***P < 0.001, ^NS^No significant difference.

In this study, we preliminarily conducted concentration gradient tests. It was found that LPS with relatively lower concentrations promoted THP-1 cell proliferation, while with higher concentrations inhibited the proliferation (Fig. 1A). On the other hand, TSA and ITSA-1 did not affect cell proliferation at the selected concentrations (Fig. 1B,C).

After the Drug intervention for 24 h, the proliferation of THP-1 cells stimulated by LPS was inhibited. The cells treated with TSA showed no significant difference in proliferation, while ITSA-1 manifestly promoted the THP-1 cell proliferation (Fig. 1D).

### TSA and ITSA-1 showed the opposite effects for LPS-induced cytokines

To mimic immunosuppression we treated THP-1 cells with 10 μg/ml of LPS with the effect of proliferation inhibition [19–21]. We found that after LPS stimulation, the TNF-α concentration increased remarkably, consistent with previous studies (Fig. 1E) [22]. At the same time, a slight upregulation of IL-6 level was observed (Fig. 1F).

Referring to the previous studies [17, 23], we used 400 nm of TSA and 50 μm of ITSA-1 to therapeutically intervene THP-1 cells. After the treatment for 24 h, the results showed that TSA significantly reduced the TNF-α level, while the further addition of ITSA-1 re-upregulated the TNF-α concentration (Fig. 1E). Concurrently, TSA had no significant effect on IL-6, while ITSA-1 slightly upregulated the IL-6 level (Fig. 1F).

### STX17 mRNA regulated by drug stimulation

**Fig. 2:**
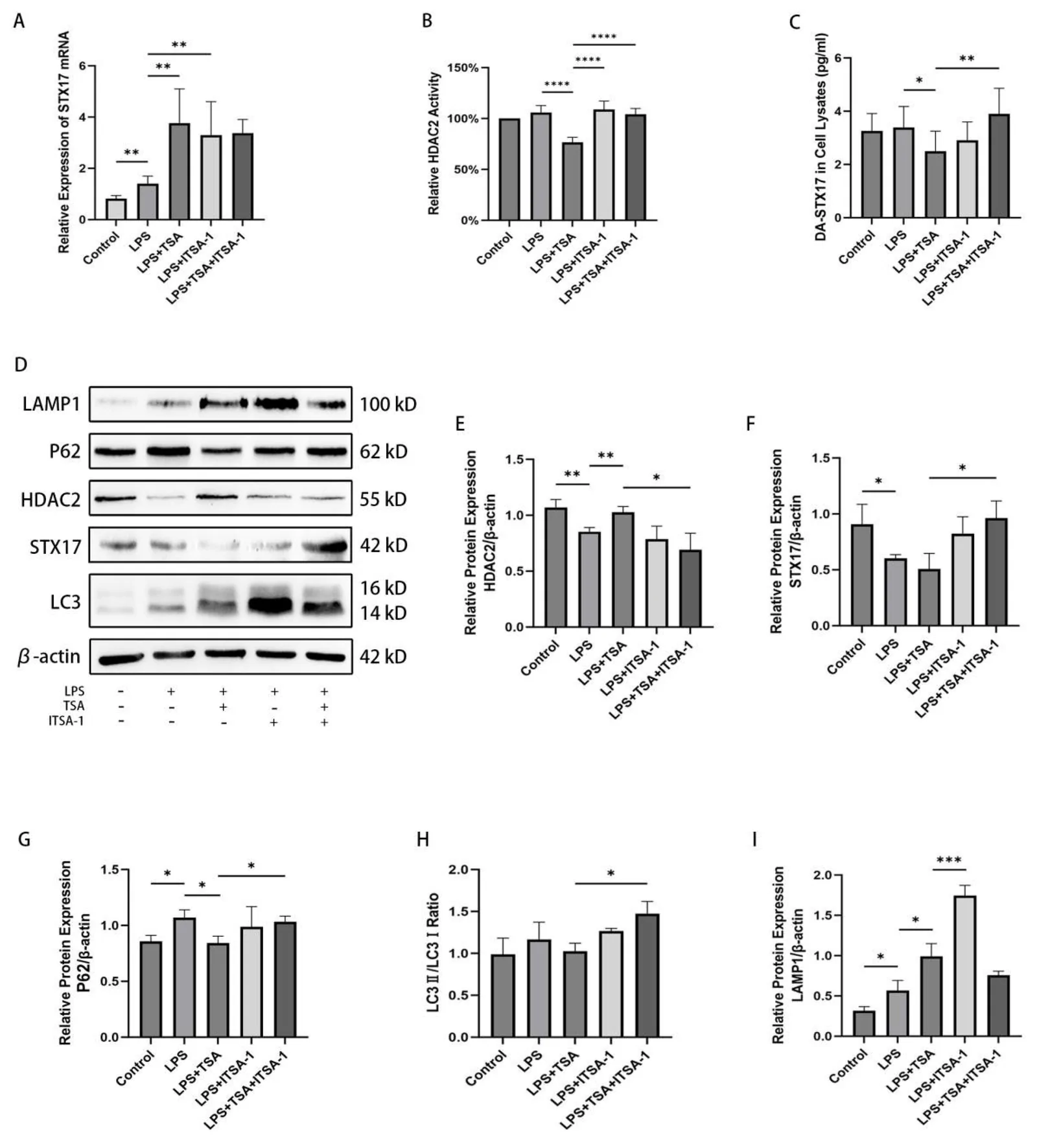
Relative expression and activity of proteins, as well as relative expression of mRNA in THP-1 cells treated with LPS, TSA, and ITSA-1 for 24 h (**A**) Protein bands gained from WB experiment (**B**) HDAC2 relative activity (**C**) Expression of DA-STX17 (**D**) Relative expression of STX17 mRNA (**E**) Relative expression of HDAC2 protein (**F**) Relative expression of STX17 protein (**G**) Relative expression of P62 protein (**H**) LC3 II/I ratio (**I**) Relative expression of LAMP1 protein. *P < 0.05, **P < 0.01, ***P < 0.001, ****P < 0.0001.

RT-qPCR was to investigate whether the alteration of the mRNA is similar to that of the protein. However, it was found that LPS slightly upregulated the STX17 mRNA expression, while TSA and ITSA-1 significantly increased the STX17 mRNA expression. The result is inconsistent with the change in STX17 protein (Fig. 2A,F).

### TSA significantly inhibited HDAC2 activity and DA-STX17 expression

In this study the results showed that after TSA intervention, HDAC2 activity was significantly decreased, while ITSA-1 reversed the effect of TSA (Fig. 2B). At the same time, DA-STX17 expression showed a synchronously positive correlation with HDAC2 activity (Fig. 2C).

### The alterations in autophagy-related protein expression

In terms of autophagy-related proteins, this time we found that LPS downregulated HDAC2 and STX17 expression (Fig. 2D–F), while upregulated the P62 and LAMP1 expression (Fig. 2G,I). TSA upregulated HDAC2 and LAMP1 expression (Fig. 2E,I), but inhibited P62 expression (Fig. 2G), which was not as expected. Besides, ITSA-1 inhibited HDAC2 expression (Fig. 2E), promoted STX17, P62, LC3 II/I, and LAMP1 expression (Fig. 2F–I).

### TSA promoted autophagosome-lysosome fusion

mCherry-GFP-LC3B adenovirus was used to trace autophagosome-lysosome fusion. GFP fluorescence quenches due to the acidic environment within the lysosome, while mCherry fluorescence remains relying on its stability. When autophagosomes fuse with lysosomes, only free red dots can be observed appearing to indicate fused autophagosome-lysosome.

**Fig. 3:**
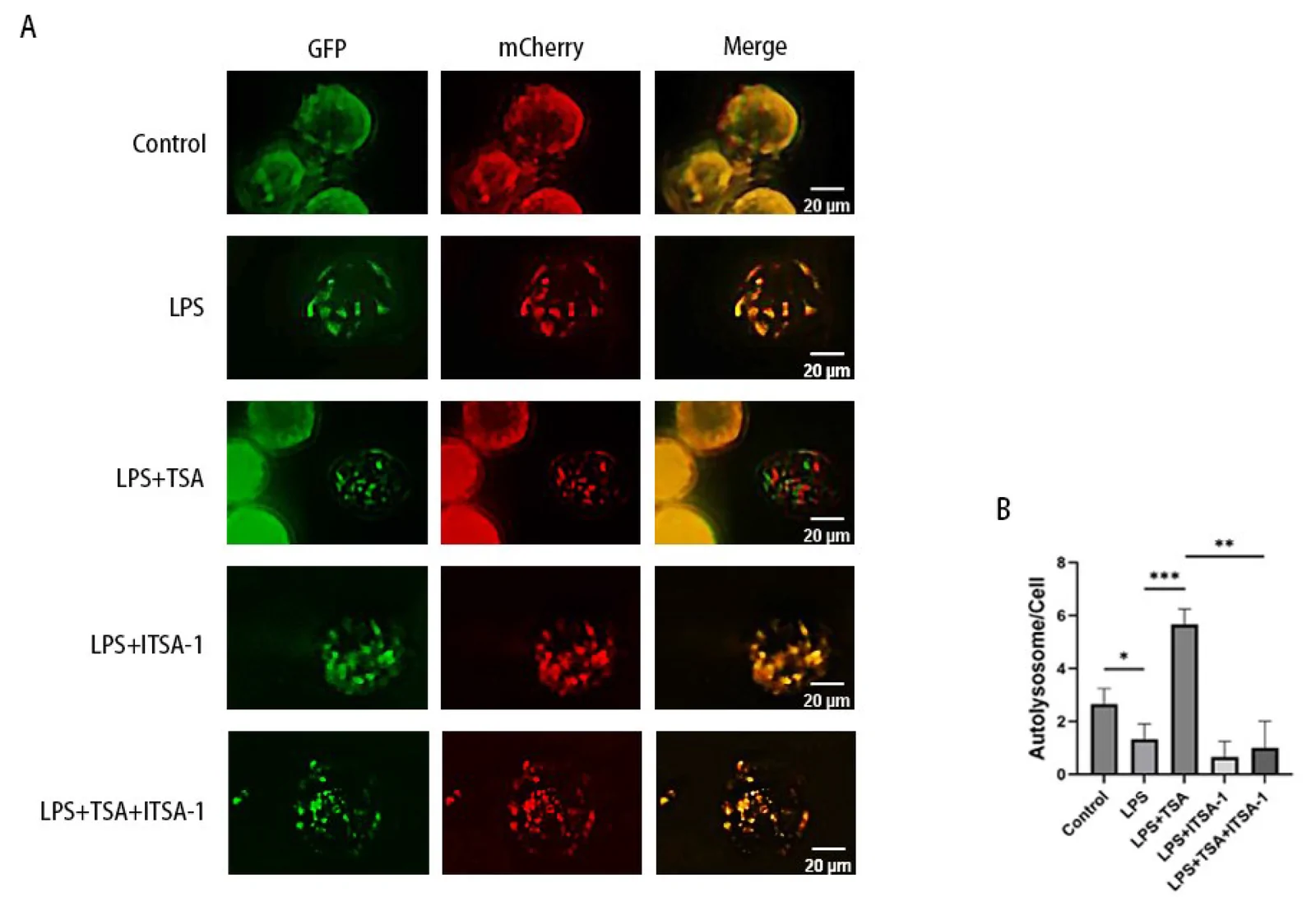
Transfection of Ad-mCherry GFP-LC3B fluorescent adenovirus in THP-1 cells intervened with LPS, TSA, and ITSA-1 for 24 h (**A**) Fluorescence test tracing autophagosome-lysosome fusion in THP-1 cells intervened with LPS, TSA, and ITSA-1 for 24 h (**B**) The amount of autophagic lysosomes measured from the fluorescence test. *P < 0.05, **P < 0.01, ***P < 0.001.

It was shown that after LPS intervention, autophagosome-lysosome fusion decreased. TSA intervention enhanced autophagosome-lysosome fusion presented as risen free red dots. Under ITSA-1 intervention, a significant reduction in free red dots was observed, reflecting a remarkable decrease in autophagosome-lysosome fusion (Fig. 3A,B).

### ITSA-1 significantly induced THP-1 cell apoptosis

**Fig. 4:**
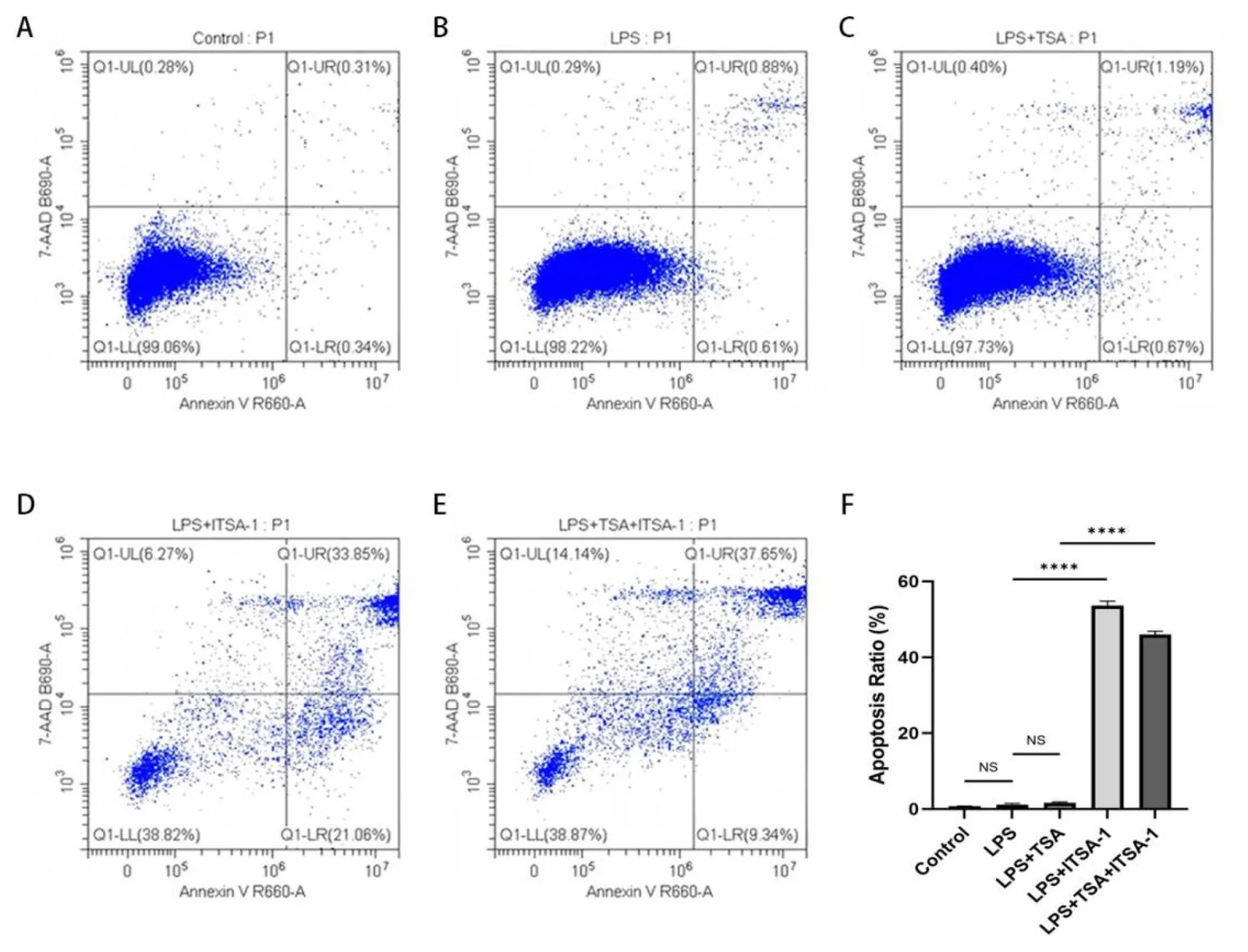
Apoptosis levels in THP-1 cells administrated with LPS, TSA, and ITSA-1 for 24 h (**A**) Cell apoptosis level in control group (**B**) Cell apoptosis level with LPS intervention (**C**) Cell apoptosis level with LPS and TSA intervention (**D**) Cell apoptosis level with LPS and ITSA-1 intervention (**E**) Cell apoptosis level with LPS, TSA, and ITSA-1 intervention (**F**) Proportions of apoptotic cells. ****P < 0.0001, ^NS^No significant difference.

We further completed flow cytometry to figure out the relation between cell apoptosis and autophagy. This time there were no significant changes in the apoptosis level of THP-1 cells stimulated with LPS and TSA (Fig. 4A–C,F). On the contrary, significant apoptosis was found in THP-1 cells after the administration with ITSA-1 (Fig. 4D–F).

### Network pharmacology analysis revealed potential mechanisms of TSA in treating sepsis

**Fig. 5:**
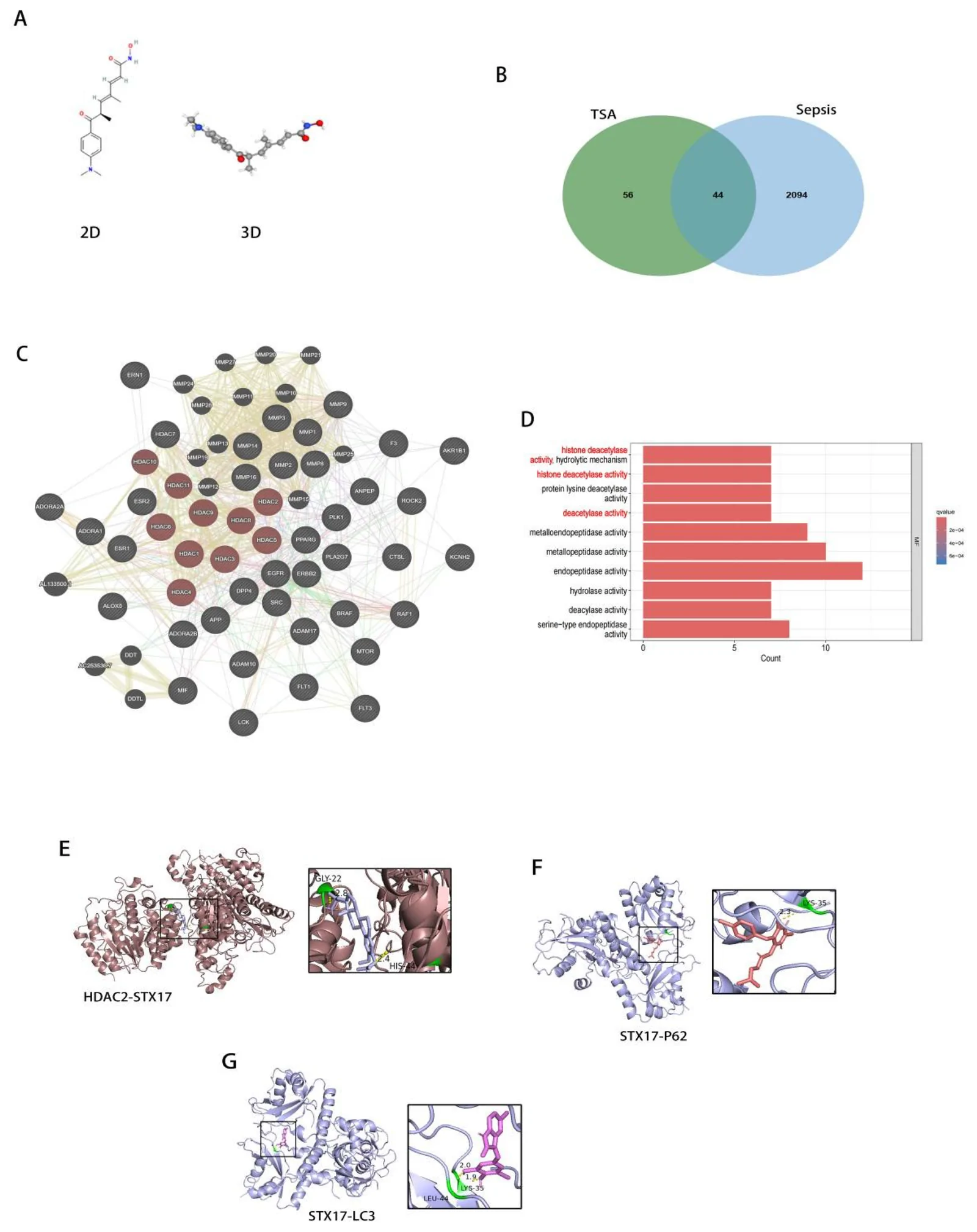
Potential targets and mechanisms of TSA in the sepsis treatment (**A**) 2D and 3D structures of TSA (**B**) Intersection targets between TSA-targeted genes and sepsis-related genes obtained by the Venn diagram (**C**) PPI network of intersection protein targets (**D**) Top 10 significantly enriched molecular functions (MF) identified via GO analysis (**E**) Molecular docking analysis of HDAC2 and STX17 (**F**) Molecular docking analysis of STX17 and P62 (**G**) Molecular docking analysis of STX17 and LC3.

Network pharmacology analysis was conducted to auxiliarily verify the reliability of utilizing TSA to cure sepsis. The 2D and 3D structures (PubChem CID: 444732) of TSA were presented in Fig. 5A. And the Veen diagram showed 42 overlapping genes which were considered potential curing targets (Fig. 5B). The PPI network found that HDAC family occupied a relatively crucial position in protein intersection (Fig. 5C). At the same time, by GO analysis histone deacetylation was revealed to play a crucial role in the interaction between TSA and sepsis (Fig. 5D). Besides, molecular docking indicated that there was excellent binding among HDAC2, STX17, and P62/LC3 (Fig. 5E–G).

## Discussion

Sepsis is an infection-induced immune-related disorder accompanied by life-threatening symptoms. Due to its complex mechanism, sepsis is difficult to treat, posing significant challenges to clinical practice.

Previously, immune hyperfunction was considered the fundamental cause of death in sepsis patients. However, it has been found that those patients who survive during the course of sepsis may subsequently develop PICS, which leads to a high rate of delayed death [7]. Notably, at the late stage of sepsis patients show a significant reduction in immunocytes [9], indicating that immunosuppression is a fundamental factor of poor prognosis.

Autophagy is a highly conserved bioprocess that stabilizes cellular homeostasis and energy recycling. However, the role of autophagy remains controversial in pathological states. Studies have shown that autophagy positively impacts sepsis. For instance, our previous experiments confirmed that doxycycline promoted autophagy, and alleviated inflammation [22]. Conversely, autophagy may also contribute to the exacerbation of sepsis under certain conditions. Excessive autophagy can impair autophagic flux, leading to autophagic cell death and enhancing inflammatory response.

During the course of sepsis, autophagy exhibits dynamic changes. It can be inferred that at the early stage of sepsis, autophagy exerts a protective effect by sustaining normal cellular function. In the late stage, immunosuppression exacerbates the disease due to weakened autophagy. Currently, studies commonly focus on the early stage of autophagy. Therefore, research concerning the late stage of autophagy may contribute to comprehensively understanding autophagy and provide novel ideas for the treatment of sepsis.

In autophagosome formation, LC3 is converted from LC3-I to LC3-II. It is generally believed that LC3-II/I ratio can reflect the level of autophagy. In addition, studies have shown that P62 expression increases when autophagosome-lysosome fusion is obstructed [14].

The late stage of autophagy is mediated by STX17. Previous studies have shown that HDAC2 deacetylates STX17, and DA-STX17 promotes autophagosome-lysosome fusion [15]. On the contrary, acetylated STX17 (A-STX17) impairs autophagosome-lysosome fusion with the accumulation of LC3. Additionally, the colocalization of LAMP1 and LC3 is also involved in the late stage of autophagy, and is positively correlated with STX17 deacetylation [15, 16].

This time we focused on the late stage of autophagy in the inflammation cell model, aiming to find out the potential therapeutic method of sepsis.

In the study we found that different concentrations of LPS had adverse effects on THP-1 cell proliferation. And the concentration of LPS was finally chosen as 10 μg/ml to simulate immunosuppression at the late stage of sepsis, which also significantly increased TNF-α concentration. At the same time, LPS intervention upregulated P62 expression, meaning that autophagy was blocked. It was also evidenced by fluorescence assay. Besides, LPS did not have an obvious influence on THP-1 cell apoptosis. Induction of apoptosis might require higher concentrations of LPS.

**Fig. 6:**
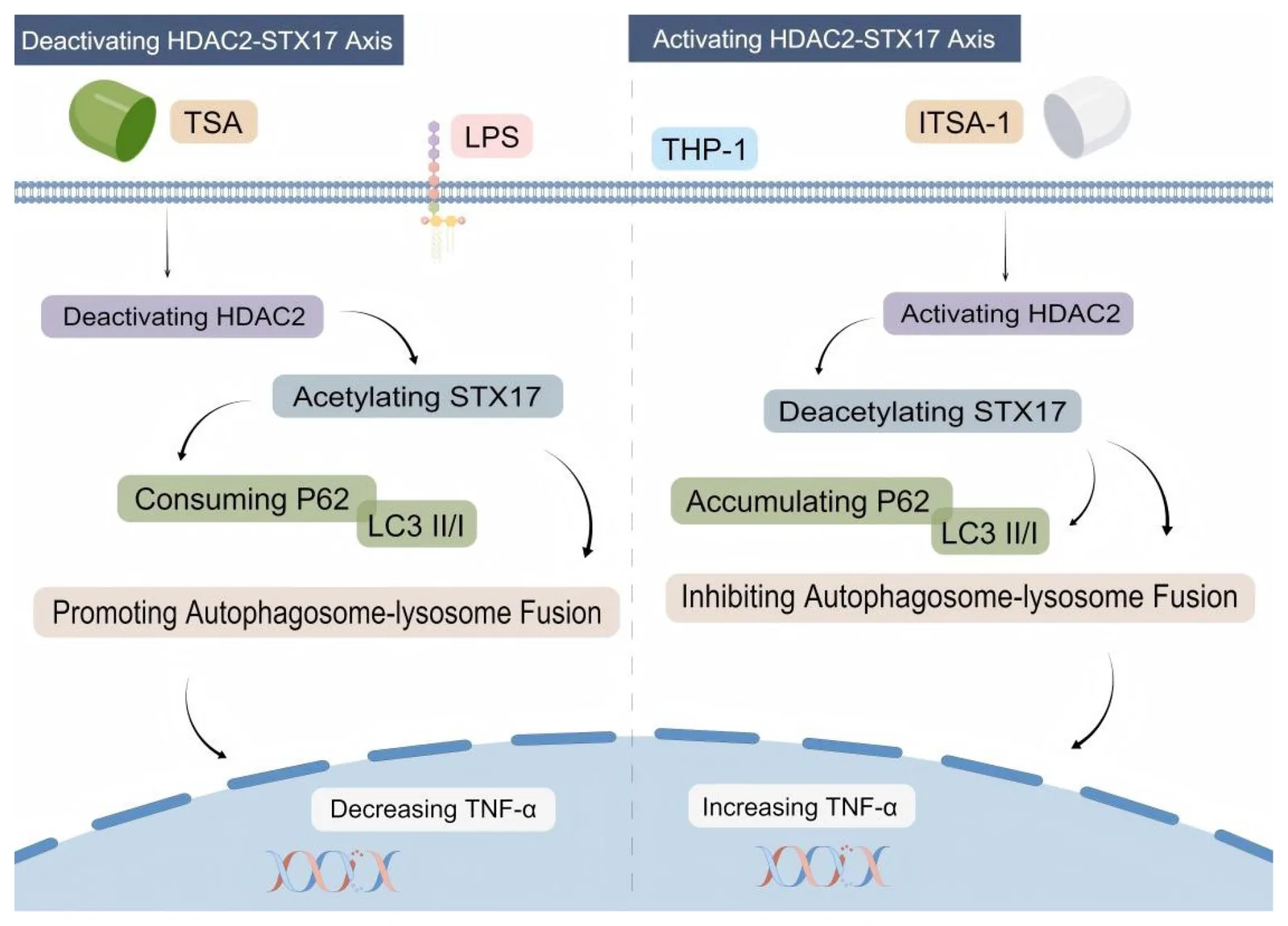
The biofunction of TSA and ITSA-1 in LPS-stimulated THP-1 cells. By Figdraw.

Concurrently, we found that TSA inhibited HDAC2 activity and downregulated DA-STX17 expression. Meanwhile, P62 expression was inhibited, indicating the late stage of autophagy was enhanced. Fluorescence assay further confirmed TSA intervention promoted autophagosome-lysosome fusion. Other studies also found that TSA reduces macrophage phenotype by enhancing autophagy, thereby alleviating inflammatory response in sepsis [24]. And TSA can alleviate sepsis-induced liver injury through the forkhead box protein O3 (Foxo3a) pathway [25]. In this study TSA similarly inhibited TNF-α secretion, without obviously impacting THP-1 cell proliferation and apoptosis (Fig. 6). What’s more, network pharmacology analysis also proved that HDAC2 is generally located at the center of the protein network, and the histone deacetylation activity regulated by HDAC is nonnegligible within the interaction between TSA and sepsis. In addition, because of the excellent binding among HDAC2, STX17, and P62/LC3 presented by molecular docking, it is credible that the autophagy mediated by the above proteins exerts certain effects in the model. Generally, a proper level of autophagy serves as anti-inflammatory effects [26, 27], in accord with the present results. Taken together, the results demonstrate the anti-inflammatory effect of TSA, and show its potential for clinical application.

On the other hand, ITSA-1 recovered HDAC2 activity and upregulated DA-STX17 expression, accompanied by an increase of P62 and LC3 II/I expression which means autophagic flux was obstructed. The block of autophagosome-lysosome fusion was also evidenced by fluorescence assay. Additionally, ITSA-1 significantly promoted TNF-α and IL-6 secretion, as well as inducing THP-1 cell proliferation and apoptosis (Fig. 6). It is believed that in the present study weakened autophagy induced by ITSA-1 exacerbated inflammatory response. Past studies also found that nonspecific inflammation was close related to enhanced cell proliferation and apoptosis [22, 28, 29]. Therefore, promotion of autophagy might be a feasible way of alleviating LPS-induced sepsis.

Notably, there were several contradictions in the research. For instance, IL-6 level did not obviously rise after LPS induction. A previous study showed that several categories of LPS only stimulated IL-6 release at 10–50 μg/ml [30]. Seemingly, in this research the significant production of IL-6 requires higher LPS concentration and longer administration time. Concurrently, TSA did not significantly change IL-6 concentration. Studies revealed that TNF-α elevated IL-6 expression via cyclic AMP response element-binding protein (CREB) binding and the acetylation of H3K14 histone marks [31]. And TSA was found to upregulate IL-6 through hyperacetylating histone H3K9 and H3K18 residues [32]. The double effects of TSA and decreased TNF-α may maintain IL-6 level relatively stable. It was found that the changes in cell proliferation and apoptosis were not completely reversed. Similar situations appeared in the previous study [33]. Alterations of cell proliferation and apoptosis may not totally comply with certain regularity. Besides, the results obtained from RT-qPCR and WB were inconsistent. The protein quantity generated from mRNA alters after several procedures such as transcription, translation, and modification. Also, temporal variations may exist in gene expression and translation towards proteins [34]. In addition, the study demonstrated that TSA intervention upregulated HDAC2 expression, whereas a previous study found that TSA inhibited HDAC2 expression in breast cancer cells [35]. Factually, there is mutual transformation between intracellular HDAC and cytoplasmic HDAC, of which expression changes under different conditions [36, 37]. The phenomenon above may play a role in LPS-induced models. Besides, P62 and LC3 expressions did not completely match. Past studies elucidated the asynchronous changes of P62 and LC3 under certain pathological conditions [38]. Moreover, several studies revealed that TSA may induce autophagy even though by the FoxO3a signal pathway or activation of the central autophagic regulator transcription factor EB (TFEB) [39, 40]. Meanwhile, TSA as HDACi has the capability of deactivating HDAC2, which can further decrease DA-STX17 expression. Reasonably, such a process inherently exists. Based on the present results, it is acceptable that TSA augments autophagy *via* downregulating DA-STX17 simultaneously. However, there has been a recognized model regarding the HDAC2-STX17 axis, which reveals that TSA impedes autophagosome-lysosome pathway[15]. Utilizations of different cell lines and intervening conditions such as administration time might lead to the aforementioned discrepancies. The previous study initiated autophagy by starvation and mTORC1 inhibition in HEK293 cells, then TSA presented an anti-autophagic effect after 16 h. While this time we found that TSA promoted autophagy after 24 h in the LPS-induced inflammatory circumstance where autophagy was preliminarily impaired. Autophagy affected by TSA might alter dynamically. Thus, the inconsistent results were obtained at different time points. Furthermore, we presume that TSA may tend to recover systems’ homeostasis, which can explain the converse performances concerning autophagy of TSA to an extent.

On the other hand, LAMP1 expression increased under all drug interventions. While after the administration with LPS and ITSA-1 which blocked autophagic flux, LAMP1 expression significantly increased. The results suggest that the excessive accumulation of late autophagy-related proteins is detrimental to autophagic flux.

A study enrolled in the GEO public database examined the STX17 gene after mechanical ventilation in LPS-intervened C57 mice. It was found that mechanical ventilation was related to the downregulation of STX17 gene, which may imply that mechanical ventilation is also associated with autophagy.

## Conclusion

In LPS-stimulated THP-1 cells, TNF-α level was remarkably elevated indicating severe inflammation in the system. Intriguingly, TSA obviously diminished the release of pro-inflammatory cytokines via inhibiting the HDAC2-STX17 axis and enhancing autophagosome-lysosome fusion. Network pharmacology analysis also provided auxiliary evidence for the objectivity of the above process. Thus, it is acceptable to use TSA to attenuate sepsis-associated diseases.

## Limitation

Admittedly, there are several aspects requiring improvements in the study. It is necessary to explore the effects of pre-administration or post-administration with the chosen drugs in further study. In addition, the interrelationships among more autophagy-related proteins need to be figured out. And *in vivo* experiments are expected to further evaluate the practicability of targeting autophagy-related proteins in therapeutics for sepsis.
